# *bkaR* is a TetR-type repressor that controls an operon associated with branched-chain keto-acid metabolism in *Mycobacteria*

**DOI:** 10.1111/1574-6968.12196

**Published:** 2013-07-08

**Authors:** Ricardo JC Balhana, Sade N Swanston, Stephen Coade, Mike Withers, Mahmudul Hasan Sikder, Neil G Stoker, Sharon L Kendall

**Affiliations:** 1 Department of Pathology and Pathogen Biology, The Royal Veterinary CollegeCamden, London, UK; 2 Division of Mycobacterial Research, MRC National Institute for Medical ResearchLondon, UK

**Keywords:** Rv2506, MSMEG_4718, accA1, accD1, transcriptional regulation, microarray

## Abstract

This study describes how *bkaR*, a highly conserved mycobacterial TetR-like transcriptional repressor, regulates a number of nearby genes that have associations with branched-chain keto-acid metabolism. *bkaR* (*MSMEG_4718*) was deleted from the nonpathogenic species *Mycobacterium smegmatis*, and changes in global gene expression were assessed using microarray analysis and reporter gene studies. *bkaR* was found to directly control the expression of 10 genes in *M. smegmatis*, and its ortholog in *Mycobacterium tuberculosis* (*Rv2506*) is predicted to control at least 12 genes. A conserved operator motif was identified, and binding of purified recombinant *M. tuberculosis* BkaR to the motif was demonstrated. Analysis of the stoichiometry of binding showed that BkaR binds to the motif as a dimer.

## Introduction

TetR regulators are abundant in the *Mycobacterium* genus, which includes several pathogenic species, and comprise over 30% of the total DNA-binding regulators in both *Mycobacterium smegmatis* and *Mycobacterium tuberculosis*. In *M. tuberculosis*, the few whose regulons have been described include Mce3R, a repressor that controls an operon involved in lipid metabolism (de la Paz Santangelo *et al*., 7), EthR, involved in the control of the activation of the pro-drug ethionamide (Baulard *et al*., 6) and KstR and KstR2, involved in the control of cholesterol catabolism (Kendall *et al*., 17, 18). Although many of the regulons defined to date are conserved in the environmental species *M. smegmatis*, their importance in the life style of the pathogenic species *M. tuberculosis* is clear. The Mce3R regulon has been implicated in the survival of *M. tuberculosis* in mice (Senaratne *et al*., 23) as have many of the genes in the KstR and KstR2 regulons (Hu *et al*., 16; Nesbitt *et al*., 20; Griffin *et al*., 14).

TetR regulators often bind to palindromic motifs in operators using the N-terminal end of the protein to repress transcription. In the presence of a ligand that binds to the C-terminus, the regulator is removed from the operator allowing access to RNA polymerase and transcription. Here, we focus on a mycobacterial TetR regulator, which we name *branched-chain keto-*a*cid regulator (*BkaR). This is highly conserved in both pathogenic and nonpathogenic *Mycobacteria* and is encoded by the gene *Rv2506* in *M. tuberculosis* and *MSMEG_4718* in *M. smegmatis*. BkaR was previously found to have potential associations with pathogenesis in a whole-genome transposon screen (Stewart *et al*., 24). We show that BkaR_*Msm*_ controls the expression of 10 genes likely to be involved in branched-chain keto-acid metabolism. We show relevance to *M. tuberculosis* through protein-binding experiments, regulatory motif analysis and reporter gene assays. The potential role of the BkaR regulon is discussed.

## Materials and methods

### Bacterial strains and culture conditions

The strains and plasmids used in this study are described in Supporting Information, Table S1 and were grown as described previously (Kendall *et al*., 18).

### Microarray analysis of *M. smegmatis ΔbkaR*_*Msm*_

Δ*bkaR*_Msm_ was available from a previous study (Balhana *et al*., 5). RNA from both wild-type and Δ*bkaR*_Msm_ strains was prepared by direct sampling into guanidine thiocyanate (GTC) followed by the use of the RNAeasy kit (Qiagen) as previously described (Kendall *et al*., 18). Microarrays were provided by the Pathogen Functional Genomics Resource Centre at TIGR (http://pfgrc.jcvi.org). cDNA from wild-type and mutant strains were labelled and competitively hybridized onto the arrays. All methods including scanning, data analysis and significance criteria were as previously described (Kendall *et al*., 18). Fully annotated microarray data have been deposited in BμG@Sbase (accession number E-BUGS-116; http://bugs.sgul.ac.uk/E-BUGS-116) and also ArrayExpress (accession number E-BUGS-116).

### RT-PCR for analysis of operon structure

RNA isolated from the Δ*bkaR*_*Msm*_ mutant was treated twice with DNase I (Invitrogen) (30 min at 37 °C). The reaction was inactivated at 65 °C for 10 min in the presence of 1.25 mM EDTA. DNA-free RNA (150 ng) was mixed with 300 ng of random primers, 10 mM DTT, 0.5 mM dNTPs and reversed-transcribed with 200 units of Superscript III reverse transcriptase (Invitrogen) according to the manufacturer's instructions. Control reactions were performed without reverse transcriptase. PCR was carried out using Phusion® High-Fidelity PCR Master Mix with GC buffer (New England Biolabs).

### Reporter analysis

This was performed using the *lacZ* integrative reporter construct pEJ414 (Papavinasasundaram *et al*., 22). Upstream intergenic regions of genes of interest were either PCR-amplified or synthesized as oligonucleotides (Table S2), cloned using NotI and XbaI and electroporated into wild-type and *ΔbkaR*_*Msm*_ strains. Reporter assays were carried out as described previously (Papavinasasundaram *et al*., 22).

### Expression and purification of recombinant BkaR_Mtb_

*bkaR*_Mtb_ was PCR-amplified from *M. tuberculosis* H37Rv genomic DNA (Table S2) and inserted into pNIC28-Bsa4 (GenBank accession no. EF198106) using ligation-independent cloning with T4 DNA polymerase. The final construct, pNbkaR-MTB was transformed into *Escherichia coli* BL21(DE3). Expression and induction were achieved in 400 mL of autoinducing medium (Studier, 25) at 37 °C overnight. Cultures were harvested by centrifugation (30 min, 3070 ***g***, 4 °C), and the pellet was resuspended in 5 mL of lysis buffer (70 mM HEPES, 20 mM imidazole, 650 mM NaCl, 0.5 mM β-mercaptoethanol, 10% glycerol, pH 8). Cells were lysed on ice using sonication (Soniprep 150) at 20 μm for 5 min with 30-s rest period every 1 min. Soluble fractions were isolated through centrifugation (11 337 ***g***, 20 min, 4 °C), and His_6_-BkaR_Mtb_ was purified using immobilized metal ion affinity chromatography on HisTrap FF Ni-Sepharose columns (GE Healthcare Life Sciences). The protein was eluted with histidine elution buffer (250 mM histidine, 60 mM HEPES, 150 mM NaCl, 3% glycerol, pH 8).

### Electrophoretic mobility shift assays

DNA oligonucleotides or PCR amplicons were used to assay the binding of His_6_-BkaR_Mtb._ Probes used for competition assays were end-labelled with DIG-11-ddUTP using the DIG gel shift kit, 2nd generation (Roche). Binding reactions were performed by incubating varying concentrations of protein with 0.03 pmol of labelled probe in 1X binding buffer (20 mM HEPES, 1 mM EDTA, 10 mM (NH_4_)_2_SO_4_, 1 mM DTT, 0.2% Tween-20, 30 mM KCl, pH 7.6) together with 0.1 μg of poly-l-lysine and 1 μg of poly[d(I-C)] in a total volume of 20 μL. Specific competitor (nonlabelled probe) was added in 150-fold excess. Mixtures also contained an excess 125-fold poly [d(I-C)] (nonspecific competitor). Reactions were incubated, separated by polyacrylamide gel electrophoresis and blotted onto Hybond-N nylon membranes as previously described (Kendall *et al*., 18). The membrane was washed according to the manufacturer's instructions and detection followed the chemiluminescent method using anti-DIG-alkaline phosphatase and the substrate CSPD. The luminescent membranes were exposed to X-ray film for varying time points between 5 min and 1 h.

### Determination of the binding stoichiometry of the His_6_–BkaR_Mtb_–DNA complex

A Ferguson plot assay was used to determine the molecular weight of the protein–DNA complex (Orchard & May, 21). Electrophoretic mobility shift assays (EMSA) reactions were run alongside protein standards through a series of gels differing in acrylamide concentration (range of 6–12%). Gels were run at 10 V cm^−1^ in 0.4X TBE buffer, and once finished, the distance migrated by the bromophenol blue was measured for each sample. Each gel was cut in half, isolating the tracks containing the protein–DNA complex from the tracks with protein standards. The gel fragments containing DNA were stained for 30 min at room temperature in 0.4X TBE with 0.68 μg mL^−1^ ethidium bromide, whereas the gel fragments containing the protein standards were Coomassie-stained (30% methanol, 8% acetic acid, 0.25% w/v Coomassie blue *R*). The distances migrated by the protein–DNA complexes and by each standard were measured and divided by the distance migrated by the bromophenol blue, giving the relative mobility (*R*_f_) for each species. The logarithms of the relative mobilities were plotted against gel concentration, and the retardation coefficients (*K*_r_, slopes of the trend curves) were calculated and plotted again vs. the molecular weight of each standard. The Ferguson plot obtained allowed determination of the molecular weight of the complex.

### Bioinformatic analyses

Genome sequences were compared using Artemis Comparision Tool (ACT) (Carver *et al*., 106), and using clustalw (Thompson *et al*., 26), operator motif discovery and analysis were carried out using MEME and MAST (Bailey & Elkan, 3; Bailey & Gribskov, 4).

## Results

### Genomic analyses of the *bkaR* region show conservation in *Mycobacteria* and closely related species

**Figure 1:**
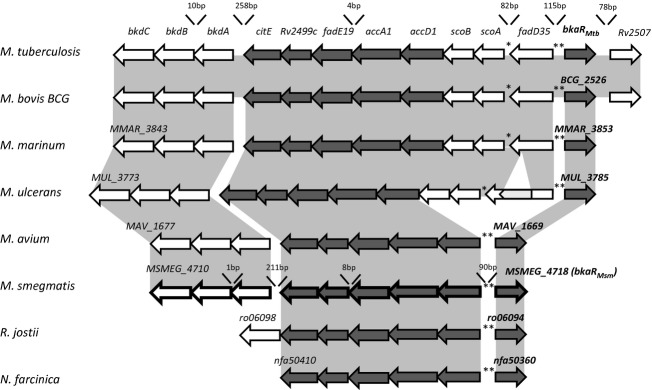
Genomic context of *bkaR* in *Mycobacteria* and close relatives. The grey shading represents regions of conservation between genomes. Arrows represented with a dark grey background correspond to genes that are conserved amongst all species, whereas genes that find no ortholog in at least one species are shown with white arrows. The light grey arrow inserted in *MUL_3785* represents a transposon insertion. Asterisks represent binding motifs identified by the programs MEME and MAST, and the sizes of the intergenic gaps in *Mycobacterium smegmatis* and *Mycobacterium tuberculosis* are indicated. Genes that were derepressed in the *ΔbkaR*_M__sm_ mutant are highlighted by thickened lines.

Using genomic alignment, *bkaR* was found to be clearly conserved in *M. tuberculosis*, *M. bovis* BCG, *Mycobacterium marinum*, *Mycobacterium ulcerans*, *Mycobacterium avium*, *M. smegmatis*, *Rhodococcus jostii* and *Norcardia farcinica* (Fig. 1). The percentage amino acid sequence identities between BkaR in *M. tuberculosis* and its orthologs were > 50% in all cases. Most neighbouring genes were also conserved, although *fadD35*, *scoA* and *scoB* were only present in species more closely related to *M. tuberculosis*.

### BkaR is autoregulatory and binds to a conserved palindromic motif within its own promoter

**Figure 2:**
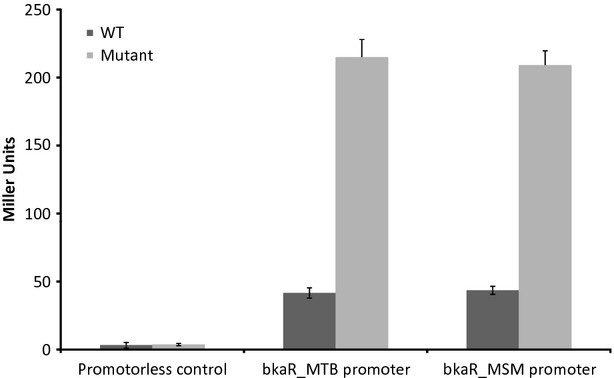
Expression of the *bkaR*_*Mtb*_ and *bkaR*_Msm_ promoter in wild-type mc^2^ 155 and *ΔbkaR*_Msm_ mutant background. Beta-galactosidase activity was calculated in Miller Units (measured as the amount of *O*-nitrophenol produced (nmol) over time (min) per mg of protein). Deletion of *bkaR*_*Msm*_ causes a statistically significant increase in the expression of *lacZ* from the *bkaR*_*Mtb*_ and the *bkaR*_Msm_ promoters (unpaired Student's *t*-test; *P* < 0.05, *). The values result from means of biological triplicates and technical duplicates (this experiment was repeated independently with similar results). Error bars represent standard deviations.

TetR regulators are generally autoregulatory. Measurement of expression from the *bkaR*_Msm_ and *bkaR*_Mtb_ promoters in *lacZ* reporter constructs in wild-type *M. smegmatis* and Δ*bkaR*_Msm_ showed that transcription was approximately fourfold higher in the strain lacking the *bkaR* regulator, indicating that both *bkaR*_Mtb_ and *bkaR*_Msm_ are autoregulatory and repress their own expression (Fig. 2).

**Figure 3:**
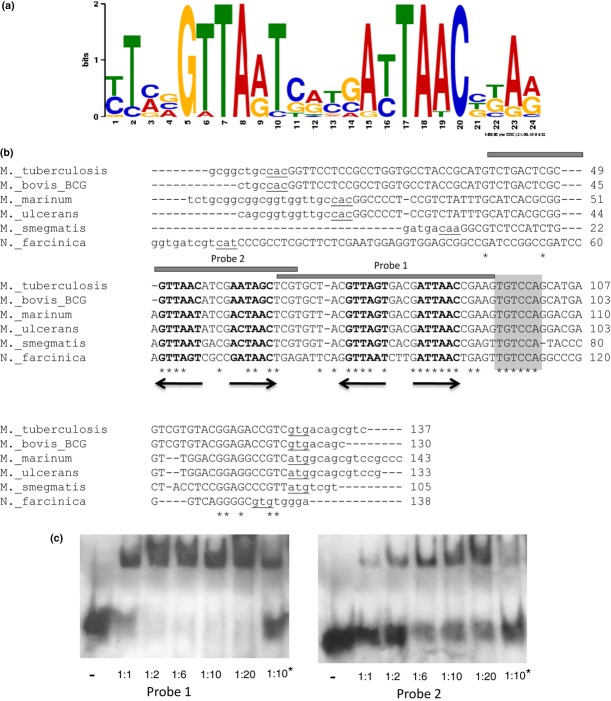
(a) Sequence logo of the putative binding motif constructed from the intergenic regions of *bkaR* orthologs in *Mycobacteria* and close relatives. Sequence logos illustrate the relative frequency of each nucleotide in a given position. The *E*-value of the motif is 1.6e^−20^, this is an estimate of the number of motifs (of equal or higher interest) expected to be found by chance if the letters in the input sequences were shuffled. (b) ClustalW alignment of the *bkaR*_*Mtb*_*/fadD35* (*bkaR*_*Msm*_/*accD1* for *Mycobacterium smegmatis*) intergenic region. Asterisks indicate conservation of residues in all genomes. An inverted palindrome is observed in two copies in all species and is represented in bold and with arrows. The location of the probes used in the EMSAs is illustrated above the sequence. Lower-case letters represent coding sequences of the two divergently oriented genes for each instance, and start codons are underlined for each gene. The area shaded in grey is a putative −35 site. (c) EMSA showing specific binding of His_6_-BkaR_M__tb_ to DIG-labelled probes 1 and 2 in the presence of 125-fold excess of the nonspecific competitor poly[d(I-C)]. DNA was incubated with protein in a variety of molar ratios as labelled at the bottom of each lane. − without protein. An asterisk shows the lanes where specific competition with 150-fold unlabelled probe took place (these lanes should be compared with the respective molarity ratio without specific competition).

Transcriptional repressors of the TetR family tend to bind to palindromic DNA motifs (Yu *et al*., 29). Computational analysis of the *bkaR* promoter using MEME identified a 24-bp palindromic motif with a highly conserved 16-bp core **GTTA**(N)**T**(N4)**A**(N)**TAAC** that was present twice in the promoter regions of all the species tested (Fig. 3a). The promoter regions of some of the *Mycobacteria* and *Nocardia* species were aligned to identify the position of the motif (Fig. 3b). Both copies of the motif were clearly visible with the 16-bp core being more conserved than the rest of the intergenic region. Additionally, there was another region that showed more conservation. This could possibly be the −35 site as it is a short conserved GC-rich region as seen in other mycobacterial promoters (Gomez & Smith, 13).

To test if BkaR binds to the two motifs, 30-bp double-stranded probes containing the motifs were used in EMSAs. The probes were DIG-labelled, and the binding assays were carried out in the presence of 125-fold excess of nonspecific competitor poly[d(I-C)] and with specific competition with unlabelled probe (Fig. 3c). Clear shifts were seen in the presence of the nonspecific competitor at all molar ratios. At a 1 : 10 molar ratio of DNA/protein, the presence of excess unlabelled probe successfully competed with the labelled probe (lanes marked with an asterisk). BkaR appears to show lower affinity for probe 2 (motif near *fadD35* in *M. tuberculosis* and *accD1* in *M. smegmatis*) as a complete shift was not achieved for any of the DNA/protein ratios, whereas with probe 1, a complete shift was observed at a 1 : 2 molar ratio.

### BkaR binds to the motif as a dimer

The stoichiometry of binding of BkaR_Mtb_ with the motif DNA was examined using Ferguson plot analysis (Ferguson, 10; Orchard & May, 21). The logarithms of the relative mobility (*R*_f_) of protein standards and BkaR_Mtb_–DNA complex were plotted against the percentage gel concentration (Data S1), and the slopes (retardation coefficient) were calculated. The retardation coefficients were subsequently plotted as a function of molecular weight. Using the equation of the adjusted curve obtained, we estimated the molecular weight of the complex to be 76.9 kDa. Subtracting the molecular weight of the 30-bp DNA oligonucleotide used (18.4 kDa), the mass accounted for the protein alone is 58.5 kDa, which approximately corresponds to the mass of an His_6_–BkaR_Mtb_ dimer (monomer is 25.8 kDa).

### Genomewide analysis of the presence of the motif in *M. smegmatis* and *M. tuberculosis* and global gene expression analyses

Computational analyses for further instances of the motif using MAST showed that there were four more instances of the motif in the *M. smegmatis* genome and three more instances in *M. tuberculosis* (Table 1). In *M. smegmatis*, these were found upstream of *MSMEG_4920, MSMEG_4524*and between the divergently oriented *MSMEG_3414/MSMEG_3415* (two copies). However, the significances of these motifs were much lower than the motif originally found within the *bkaR* promoter. DNA-binding studies provided experimental corroboration for the motif upstream of *MSMEG_4920* only (Data S3).

**Table 1:** Occurrences of the motif in *Mycobacterium smegmatis* and *Mycobacterium tuberculosis*

Motif sequence	*E*-value	Flanking genes	EMSA	Reporter assay	Microarray analysis
*M. smegmatis*					
CTCG**GTTA****A****T**CGTG**A**C**TAAC**GTAC	1.4e-05	***bkaR***_**Msm**_/*MSMEG_4717*	+	+	+
CTGA**GTTA**A**T**GACG**A**C**TAAC**TCGT	3.9E-05	***MSMEG_4717/bkaR***_**Msm**_	+	ND	+
GCCG**GTTA**A**T**CATCGT**TAAC**ACAG	0.6	*MSMEG_4920*	+	ND	+
CCAG**GTTA**GCCAAA**A**C**TA**TTTCAG	2	***MSMEG_3414**/MSMEG_3415*	−	−	−
CTGAAA**TA**G**T**TTTGGC**TAAC**CTGG	3.4	*MSMEG_3414*/***MSMEG_3415***	−	−	−
CTAG**GTTA**GGCTAC**A**T**T**T**AC**TTGC	8.2	*MSMEG_4524*	−	−	−
*M. tuberculosis*					
CTAC**GTTA**G**T**GACG**A**T**TAAC**CGAA	6.1e-06	***bkaR***_**Mtb**_/*fadD35*	+	+	ND
TTCG**GTTA**ACATCG**A**A**TA**G**C**TTAG	1.1e-03	*bkaR*_Mtb_/***fadD35***	+	ND	ND
CTCA**GTTA**A**T**GATA**A**T**TAAC**TGAA	9.8E-06	*scoA*	+	ND	ND
ATAC**GTTA**GCGCTC**A**C**TAAC**GTAT	2.7	***Rv0575c**/Rv0576*	−	−	ND
ATAC**GTTA**G**T**GAGCGC**TAAC**GTAT	3.1	*Rv0575c/**Rv0576***	−	−	ND

ND, not done; +, complete shift or derepression in the case of reporter and microarray analysis; −, no shift observed or no derepression of gene expression; underlined nucleotides represent conserved base pairs of the motif; gene numbers in bold represent the ones that are closer to the motif for divergent arrangements.

In *M. tuberculosis*, the additional motifs were found upstream of *Rv2503c* (*scoA*) and between the divergently oriented *Rv0575c/Rv0576* (two copies). The putative motif upstream of *scoA* (which lies close to *bkaR* in the *M. tuberculosis* genome, but is not present in *M. smegmatis*; see Fig. 1) had comparable significance to the original motifs identified by MEME, and we were able to observe binding of purified BkaR_Mtb_ to this motif using EMSA (Data S3). However, the occurrences of the motifs in the intergenic region between *Rv0575c* and *Rv0576* were less significant, and no DNA binding was observed (data not shown). These results indicate that the motifs upstream of *MSMEG_4920* and *scoA* are bound by BkaR but those identified upstream of the other genes are either not functional binding sites or motifs for an unidentified transcriptional regulator.

Global gene expression changes as a result of deleting *bkaR* were examined by microarray analysis of wild-type and *ΔbkaR*_Msm_ strains. A total of 14 genes were found to be significantly derepressed for a *P*-value cut-off of 0.05 (Table 2). Additionally, *MSMEG_4710* (*bkdC*) was also derepressed but did not meet the significance criteria. Although this gene did not meet the significance cut-off, it was 1.8-fold derepressed, and other evidence suggests that it is part of the regulon; therefore, it was included in the table (see below).

**Table 2:** The regulon of *bkaR*_Msm_

*Mycobacterium smegmatis*	Fold change	P-value	Gene name	Ortholog in *Mtb*	Annotated function
*MSMEG_5576*	3.4	2.2E-02	–	*–*	d-mannonate oxidoreductase
***MSMEG_4920***	4.9*	7.9E-04	***fadA4***	*Rv1323*	Acetyl-CoA acetyltransferase
***MSMEG_4718***	−1.8*	4.8E-01	***bkaR***	*Rv2506*	TetR transcriptional regulator (***bkaR***)*
***MSMEG_4717***	9.0*	3.7E-05	***accD1***	*Rv2502c*	Acetyl/propionyl-coenzyme A carboxylase (β subunit)
***MSMEG_4716***	15.0	1.5E-05	***accA1***	*Rv2501c*	Acetyl/propionyl-coenzyme A carboxylase (α subunit)
***MSMEG_4715***	13.7	5.8E-06	***fadE19***	*Rv2500c*	Acyl-CoA dehydrogenase
***MSMEG_4714***	9.5	1.1E-04	***Rv2499c***	*Rv2499c*	Hydratase
***MSMEG_4713***	16.5	4.6E-06	***citE***	*Rv2498c*	HpcH/HpaI aldolase/citrate lyase family protein
***MSMEG_4712***	8.4	1.1E-04	***bkdA***	*Rv2497c*	Part of branched-chain keto-acid dehydrogenase complex†
***MSMEG_4711***	3.3	3.3E-02	***bkdB***	*Rv2496c*	Part of branched-chain keto-acid dehydrogenase complex*
***MSMEG_4710***	1.8	4.9E-01	***bkdC***	*Rv2495c*	Part of branched-chain keto-acid dehydrogenase complex*
*MSMEG_4005*	−3.2	2.5E-02	*–*	*–*	Calcium-binding protein
*MSMEG_2080*	3.1	3.3E-02	*fadE23*	*Rv3140*	Putative acyl-CoA dehydrogenase
*MSMEG_1885*	3.3	2.2E-02	*Rv3230c*	*Rv3230c*	Iron–sulphur cluster binding domain protein
*MSMEG_1548*	3.0	4.3E-02	*–*	*–*	Dehydratase (propanediol utilization)
*MSMEG_1543*	3.2	2.3E-02	*Rv0458*	*Rv0458*	EPTC-Inducible aldehyde dehydrogenase
*MSMEG_0881*	−3.4	2.2E-02	*–*	*–*	Hypothetical protein
*MSMEG_0066*	3.3	2.2E-02	*esxA*	*Rv3875*	Early secretory antigenic target

The genes in bold are those directly regulated by *bkaR*.

* Occurrence of the motif.

† Venugopal *et al*. (28).

The highest fold change (> 8) was observed in genes that are part of the operon divergently oriented to *bkaR*_Msm_ (*accD1-citE*) (Fig. 1). With the exception of an 8-bp gap between *fadE19* and *MSMEG_4714,* the genes *accD1-citE* are contiguous and are likely to be expressed as a single transcript. Therefore, the microarray data suggest that, as predicted by the presence of the motif, the regulator controls the expression of the divergently transcribed genes between *accD1-citE* in *M. smegmatis*. This correlates with the observation that TetR regulators often control adjacent genes (Ahn *et al*., 1).

The genes between *bkdA* and *bkdC* were also derepressed in the mutant (although the last gene *bkdC* was not significant at the chosen *P*-value cut-off). This was unexpected because of the presence of a 211-bp gap upstream of *bkdA* with no associated regulatory motif. Therefore, RT-PCR was used to assess whether *bkdABC* were cotranscribed with the upstream genes. Reactions were performed with primers annealing across runs of genes between *fadE19* and *bkdA* incorporating the 211-bp gap (Data S2). The presence of a band of the expected size in the samples that were reverse-transcribed samples only (+) supports the microarray data and indicates that these genes are indeed cotranscribed. Large gaps (> 200 bp) have been reported to separate genes that form an operon in other bacterial species (Krause *et al*., 19). *MSMEG_4920* was also significantly up-regulated with over fourfold change and has a proximal motif to which the regulator bound (Data S3). These combined evidence strongly suggest that *MSMEG_4920* is also directly under the control of the regulator.

The remaining genes that are either significantly derepressed (*MSMEG_5576*, *MSMEG_2080*, *MSMEG_1885*, *MSMEG_1548*, *MSMEG_1543*, *MSMEG_0066*) or significantly repressed (*MSMEG_4005*, *MSMEG_0881*) are not associated with a motif. We conclude that these are not likely to be under the direct control of BkaR. The changes in expression in these genes may be an indirect effect of knocking out the regulator.

## Discussion

### The *bkaR* regulon in *M. smegmatis* and prediction of the regulon in *M. tuberculosis*

This study shows that the highly conserved transcriptional regulator *bkaR* binds to a 16-bp palindromic motif and to act as a repressor to directly control expression of itself, and of the divergently oriented operon (Fig. 1). In *M. smegmatis*, this consists of the genes from *accD1*-*bkdC*. Additionally, *bkaR*_*Msm*_ controls the expression of *MSMEG_4920,* which is de-repressed in the microarray analysis and has an associated upstream motif.

While writing this manuscript, a paper was published describing a single motif in the intergenic region of *bkaR_fadD35* in *M. tuberculosis*. Binding was demonstrated to this region together with affinity measurements, and it was shown that both tetracycline and palmityl-coA could interfere with binding (Anand *et al*., 2). These authors suggested that *bkaR* (which they call *fad35R* and consider to be a homologue of *E. coli* FadR) controls the expression of *fadD35* in *M. tuberculosis*. However, the palindrome described by Anand *et al*. was only partially identified (the authors describe a diffuse and poorly conserved palindrome), only a single motif in the *bkaR_fadD35* region was described, the motif upstream of *scoA* was not identified, and no gene expression studies were carried out to support the work. In contrast, our data clearly show the presence of two palindromic motifs in the *bkaR-fadD35* intergenic region, a motif upstream of *scoA,* and we provide gene expression data to support the identification of the regulon in *M. smegmatis*.

Gene expression studies in combination with motif analysis have previously allowed us to use data from *M. smegmatis* to predict the regulon in *M. tuberculosis*, and these predictions have been subsequently experimentally verified (Kendall *et al*., 17; Nesbitt *et al*., 20). Similarly, in this study, we predict that in addition to the orthologs shown in Table 2, *fadD35* and the cotranscribed genes *scoA-scoB* will be controlled by BkaR in *M. tuberculosis*, and we show that BkaR_Mtb_ binds to the motifs upstream of *fadD35* and *scoA*. The presence of the two motifs in the *bkaR_fadD35* intergenic region strongly suggests that *bkaR* acts like the paradigm TetR where the repressor binds as a dimer to each motif to repress expression in both directions (Hillen & Berens, 15).

### Possible functions of the genes in the *bkaR* regulon

Many of the genes in the regulon have annotated functions that could be involved in β-oxidation. However, much of the *M. tuberculosis* genome is dedicated to fatty acid β-oxidation, and it is often difficult to work out the precise substrates and exactly what role each of the enzymes play in the pathogens lifestyle. This is compounded by re-annotation of some of the genes in the regulon. The *bkdABC* genes were originally annotated as encoding for the pyruvate dehydrogenase complex (*pdhABC*), but have been found not to possess such activity (Tian *et al*., 27). More recent work suggests that they form part of a complex that has branched-chain keto-acid dehydrogenase (BCKADH) activity, which is the second stage in the catabolism of the branched-chain amino acids leucine, valine and isoleucine (Venugopal *et al*., 28).

This latter observation has led us to speculate that the other genes in the regulon also catalyse reactions in this pathway (Data S4). The branched-chain keto-acid derivatives are activated by the addition of coenzyme A (the product of BCKADH activity) and are then degraded in a series of reactions that involve dehydrogenase (*fadE19*), hydratase (*Rv2499c*), and, specifically in the case of leucine, carboxylase (*accA1accD1*) activity. The ultimate endpoint of the catabolism of branched-chain amino acids is acetyl-coA and propionyl-coA. Acetyl-coA can enter the glyoxylate cycle where it is used for energy generation, while propionyl-coA enters the methyl citrate cycle and the methyl malonyl pathway. Intermediates from these cycles can enter the TCA cycle; in this way, energy can be derived from the breakdown of branched-chain amino acids. Interestingly, other recent studies have also found evidence for the role of *accA1accD1* in branched-chain amino acid catabolism in *Mycobacteria* (Ehebaur *et al*., 9).

The branched-chain keto-acid derivatives (namely isobutyryl-CoA and 2-methyl-butyryl-CoA) also act as precursors for branched-chain fatty acid synthesis, and so, it is possible that the other genes in the *bkaR* regulon are involved in a synthetic pathway. In this alternative scenario, the *acc* genes act as other biotin-dependent carboxylases used for the synthesis of multi-methyl-branched fatty acids that can be units for mycolic acids, as in the case of *accD4* and *accD5* in *M. tuberculosis* (Gande *et al*., 11, 12; Daniel *et al*., 8).

In conclusion, we have described the regulation of a set of genes likely to be involved in branched-chain keto-acid metabolism genes in *Mycobacteria*. The genes are repressed by an adjacent autoregulatory TetR regulator, *bkaR*, which binds to a conserved palindromic motif in its own promoter region and the promoter regions of the genes it controls.

## Notes

**Table S1.** Bacterial strains and plasmids used in this study. **Table S2.** Primers and oligonucleotides used in this study. Sites used in cloning, or, in the case of EMSAs, the location of the motif, are underlined. **Data S1.** Stoichiometry of the His_6_-B kaR_Mtb_-DNA complex. **Data S2.** RT-PCR results for the run of genes *fadE19-bKDA* performed with cDNA derived from *ΔbkaR*_Msm_. **Data S3.** EMSA with purified His6-BkaR and probes containing the motifs predicted by MAST in *M.smegmatis* and *M. tuberculosis*. **Data S4.** Overview of branched chain amino acid metabolism and the possible involvement of the *bkaR* regulon.
