# Supplementary material for: bkaR is a TetR-type repressor that controls an operon associated with branched-chain keto-acid metabolism in Mycobacteria
Source: FEMS Microbiol Lett. 2013 Jul 8;345(2):132–40. doi: 10.1111/1574-6968.12196 (PMC3920632; doi:10.1111/1574-6968.12196)
Supplement: Supplementary file 1 — Table S1. Bacterial strains and plasmids used in this study. Table S2. Primers and oligonucleotides used in this study. Sites used in cloning, or, in the case of EMSAs, the location of the motif, are underlined. Data S1. Stoichiometry of the His6-B kaRMtb-DNA complex. Data S2. RT-PCR results for the run of genes fadE19-bKDA performed with cDNA derived from ΔbkaRMsm. Data S3. EMSA with purified His6-BkaR and probes containing the motifs predicted by MAST in M.smegmatis and M. tuberculosis. Data S4. Overview of branched chain amino acid metabolism and the possible involvement of the bkaR regulon. [file fml0345-0132-sd1.docx]

**S1** Bacterial strains and plasmids used in this study

| **Strain/plasmid** | **Genotype/description** | **Source** |
| --- | --- | --- |
| **Strains** |  |  |
| *E. coli* |  |  |
| DH5α | *SupE44 ΔlacU169 (lacZΔM15) hsdR17 recA1 endA1 gyrA96 thi-1 relA1* | Invitrogen |
|  |  |  |
| BL21(DE3) | *ompThsdSB(rB^–^mB^–^) gal dcm (DE3)* | Novagen |
|  |  |  |
| *M. smegmatis* |  |  |
| mc^2^ 155 | High-frequency transformation mutant ATCC 607 | (Snapper*, et al.*, 1990) |
|  |  |  |
| *ΔbkaR_Msm_* | 568 bp deletion of *MSMEG_4718* | (Balhana*, et al.*, 2010) |
|  |  |  |
| **Plasmids** |  |  |
| pNIC28-BSA4 | *E. coli* expression vector with LIC, *Kan* | Structural Genomics Consortium |
|  |  |  |
| pRB5-MSM | Δ*bkaR_Msm_*in pNILRB5 with the marker cassette from pGOAL19, *Kan, Hyg* |  |
|  |  |  |
| pNbkaR-MTB | *bkaR_Mtb_*in the expression vector pNIC28-BSA4 | This study |
|  |  |  |
| pEJ414 | Promoterless*lacZ* reporter vector for transcriptional fusions, *Kan* | (Papavinasasundaram*, et al.*, 2001) |
|  |  |  |
| pEJbkaR-MSM | *lacZ* transcriptional fusion with the upstream region of *bkaR_Msm_*in pEJ414, *Kan* | This study |
|  |  |  |
| pEJbkaR-MTB | *lacZ* transcriptional fusion with the upstream region of*bkaR_Mtb_* in pEJ414, *Kan* | This study |
|  |  |  |
| pEJ0576 | *lacZ* transcriptional fusion with the upstream region of *Rv0576* in pEJ414, *Kan* | This study |
|  |  |  |
| pEJ0575 | *lacZ* transcriptional fusion with the upstream region of *Rv0575* in pEJ414, *Kan* | This study |
|  |  |  |
| pEJ3414 | *lacZ* transcriptional fusion with the upstream region of *MSMEG_3414* in pEJ414, *Kan* | This study |
|  |  |  |
| pEJ3415 | *lacZ* transcriptional fusion with the upstream region of *MSMEG_3415* in pEJ414, *Kan* | This study |
|  |  |  |
| pEJ4524 | *lacZ* transcriptional fusion with the upstream region of *MSMEG_4524* in pEJ414, *Kan* | This study |
|  |  |  |
|  |  |  |
|  |  |  |
|  |  |  |

**S2** Primers and oligonucleotides used in this study. Sites used in cloning, or, in the case of EMSAs, the location of the motif, are underlined.

| **Region/gene of interest** | **Sequence in the 5’ to 3’ direction** | | |
| --- | --- | --- | --- |
|  |  | | |
|  | **Primers used in cloning for expression** | | |
|  | **Forward** | **Reverse** | |
| *bkaR_Mtb_* | **TACTTCCAATCCATG**ACAGCGTCCGCCCC | **TATCCACCTTTACTG**TCATAGACAACGATCCGCGC | |
|  |  | | |
|  |  | | |
|  | **Primers used in RT-PCR analyses** | | |
|  | **Forward** | | **Reverse** |
| *fadE19-bkdA* | CAGGCAAGCCGTTCAAGAAG | | GGTAGGTGACGGCCTCGAT |
|  |  | |  |
|  | **Oligonucleotides used in EMSAs** | | |
| Flanking genes/ | **Forward** | | **Reverse** |
| *bkaR_Mtb_/fadD35* (probe 1) | TCGTGCTACGTTAGTGACGATTAACCGAAG | | CTTCGGTTAATCGTCACTAACGTAGCACGA |
| *bkaR_Mtb_/fadD35* (probe 2) | TCTGACTCGCGTTAACATCGAATAGCTCGT | | ACGAGCTATTCGATGTTAACGCGAGTCAGA |
| *scoA* | CTCACCTCAGTTAATGATAATTAACTGAAA | | TTTCAGTTAATTATCATTAACTGAGGTGAG |
| *MSMEG_4920* | GCTGTGTTAACGATGATTAACCGGCACAGC | | GCTGTGCCGGTTAATCATCGTTAACACAGC |
| *MSMEG_3414* | TCGGTCCAGGTTAGCCAAAACTATTTCAGAA | | TTCTGAAATAGTTTTGGCTAACCTGGACCGA |
| *MSMEG_3415* | TACTTCTGAAATAGTTTTGGCTAACCTGGAC | | GTCCAGGTTAGCCAAAACTATTTCAGAAGTA |
| *MSMEG_4524* | CAGCAAGTAAATGTAGCCTAACCTAGCTACG | | CGTAGCTAGGTTAGGCTACATTTACTTGCTG |
| *Rv0576* | GATCAATACGTTAGTGAGCGCTAACGTATT | | AATACGTTAGCGCTCACTAACGTATTGATC |
| *Rv0575c* | ACGCCAATACGTTAGCGCTCACTAACGTATTG | | CAATACGTTAGTGAGCGCTAACGTATTGGCGT |
|  |  | |  |
|  | **PCR primers/oligonucleotides used in reporter vector cloning** | | |
| Downstream gene | **Forward** | | **Reverse** |
| *bkaR_Mtb_* | GCTCTAGACGATTGGGGTCTACGACTTC | | AATGCGGCCGCGACGGTCTCCGTACACGACT |
| *Rv0576* | CTAGATCGGCAATAGTTTAGAACAAGACCGGTCGCTCGTTGCCCCTTGATCAATACGTTAGTGAGCGCTAACGTATTGGCGTGTGCCCGACGC | | GGCCGCGTCGGGCACACGCCAATACGTTAGCGCTCACTAACGTATTGATCAAGGGGCAACGAGCGACCGGTCTTGTTCTAAACTATTGCCGATT |
| *Rv0575c* | CTAGACAATACGTTAGCGCTCACTAACGTATTGATCAAGGGGCAACGAGCGACCGGTCTTGTTCTAAACTATTGCCGAAGAGCACAATTGCAGTGC | | GGCCGCACTGCAATTGTGCTCTTCGGCAATAGTTTAGAACAAGACCGGTCGCTCGTTGCCCCTTGATCAATACGTTAGTGAGCGCTAACGTATTGT |
| *bkaR_Msm_* | CGTCTAGAGTCGCGATGCGATGACAAG | | TAAGCGGCCGCAACGGGCTCCGGAGGTAGG |
| *MSMEG_3414* | CTAGACGTCGTCCATCGGTCCAGGTTAGCCAAAACTATTTCAGAAGTAAAACAGTGATAAAGTTTCCCGGTGCTTGCTTCCCCTGC | | GGCCGCAGGGGAAGCAAGCACCGGGAAACTTTATCACTGTTTTACTTCTGAAATAGTTTTGGCTAACCTGGACCGATGGACGACGT |
| *MSMEG_3415* | CTAGAGAAACTTTATCACTGTTTTACTTCTGAAATAGTTTTGGCTAACCTGGACCGATGGACGACGCAGCGGACGACGTTGC | | GGCCGCAACGTCGTCCGCTGCGTCGTCCATCGGTCCAGGTTAGCCAAAACTATTTCAGAAGTAAAACAGTGATAAAGTTTCT |
| *MSMEG_4524* | CTAGAGAACGGCGTGGTGCACCTTTCGTAGCTAGGTTAGGCTACATTTACTTGCTGGC | | GGCCGCCAGCAAGTAAATGTAGCCTAACCTAGCTACGAAAGGTGCACCACGCCGTT |

**S1. Stoichiometry of the His_6_-BkaR_Mtb_-DNA complex**.**(A)** Calibration curves plotted with the logarithm of the relative mobility of standard proteins and BkaR_Mtb_-DNA complex against acrylamide gel concentration. **(B)** Ferguson Plot showing the retardation coefficient (negative slope) plotted as a function of molecular weight of proteins.The value corresponding to the protein-DNA complex is indicated with a vertical arrow. The equations of the adjusted curves for each species are shown in the respective graph.

(A)

(B)


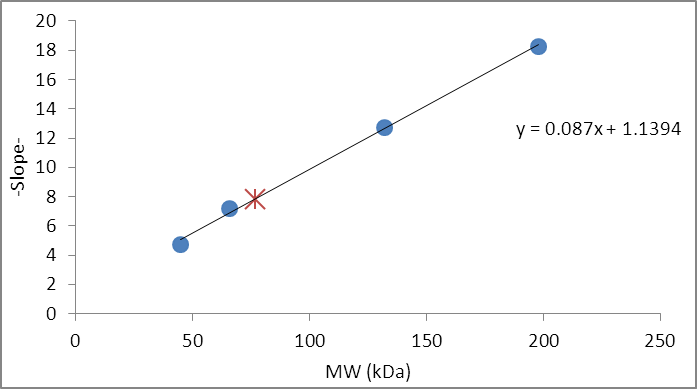


DNA/protein complex


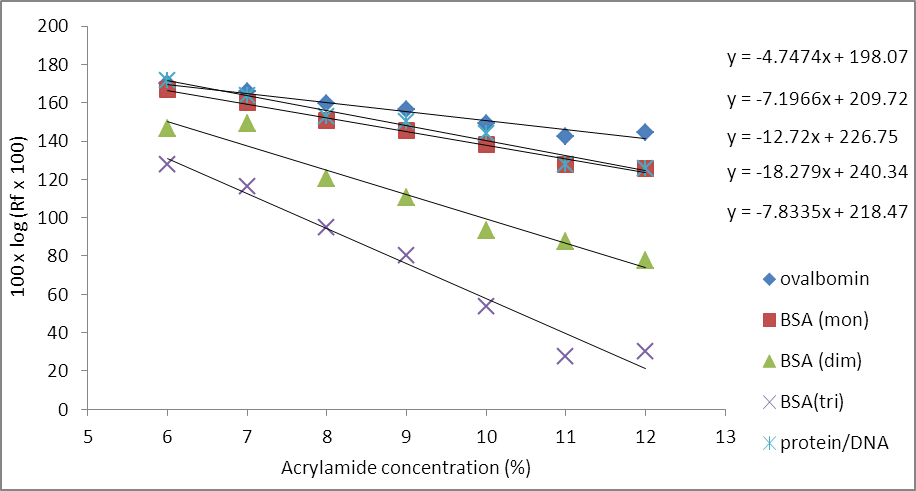

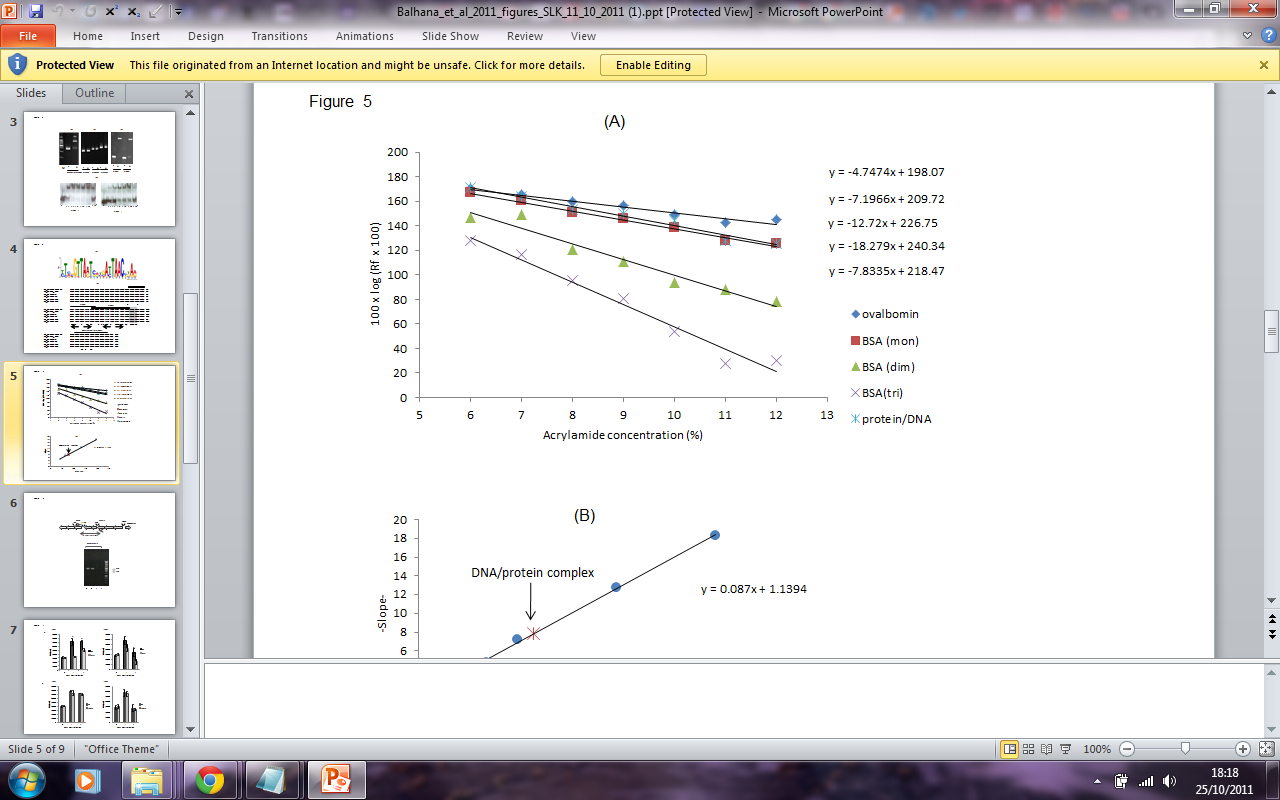


**S2. RT-PCR results for the run of genes *fadE19-bkdA* performed with cDNA derived from *ΔbkaR*_Msm_.** -, without reverse transcription; +, with reverse transcription. Two technical duplicate reactions were run together for the RT+ and RT- samples. The presence of a band reveals co-transcription of the *M. smegmatis* target genes. The approximate sizes of each PCR product are indicated in the schematic and one-ended arrows show where the primers anneal for each product.

**+ + - -**


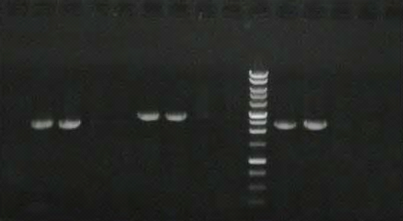


*bkdA-fadE19*

***bkaR_Msm_***

**

8bp

211bp

1bp

90bp

2.5 kb transcript

**3 kb**

**2 kb**

*bkdA*

*fadE19*

(A)

(B)

+

-

+

-

**S3. EMSA with purified His6-BkaR and probes containing the motifs predicted by MAST in *M. smegmatis*and *M. tuberculosis*.** Both themotifs upstream of *scoA*(in *M. tuberculosis*) and *MSMEG_4920* (in *M.smegmatis*) show a positive shift. Protein was incubated with DNA in a ratio of10:1 (0.6 μM of protein:0.06μM of DNA). +, with protein; - without protein.


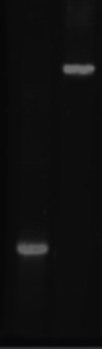

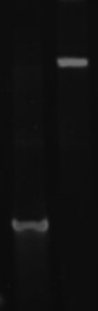


*scoA*

*MSMEG_4920*

+

-

+

-

**S4 Overview of branched chain amino acid metabolism and the possible involvement of the *bkaR*regulon.** The *bkdABC*genes,which are controlled by*bkaR,* function as branched-chain keto acid dehydrogenase (BCKADH) in complex with Lpd. The fate of the products of BCKADH activity can be used for energy generation or for the synthesis of branched chain fatty acids. The remaining genes in the *bkaR*regulon could function in either fate.

**Leucine**

**Isoleucine**

**Valine**

**Ketoleucine**

**Ketoisoleucine**

**Ketovaline**

**Acetyl-CoA**

**Propionyl-CoA**

***bkdA***

***bkdB****+ lpd*

***bkdC***

**Isovaleryl-CoA**

**2-Methylbutryl-CoA**

**Isobutryl-CoA**

**Methyl-branched chain fatty acids**

***fadE19, Rv2499c, accA1/accD1?***

***fadE19, Rv2499c, accA1/accD1?***

Balhana R, Stoker NG, Sikder MH, Chauviac FX & Kendall SL (2010) Rapid construction of mycobacterial mutagenesis vectors using ligation-independent cloning.*J Microbiol Methods***83**: 34-41.

Papavinasasundaram KG, Anderson C, Brooks PC, Thomas NA, Movahedzadeh F, Jenner PJ, Colston MJ & Davis EO (2001) Slow induction of RecA by DNA damage in Mycobacterium tuberculosis. *Microbiology***147**: 3271-3279.

Snapper SB, Melton RE, Mustafa S, Kieser T & Jacobs WR, Jr. (1990) Isolation and characterization of efficient plasmid transformation mutants of *Mycobacterium smegmatis*. *MolMicrobiol***4**: 1911-1919.
